# Structural Evolution in Tin Chloride through Neutron Irradiation: Toward Indium-Doped Tin

**DOI:** 10.1021/acsomega.5c01603

**Published:** 2025-05-16

**Authors:** Rodrigo F.B. de Souza, Gabriel Silvestrin, Edson P. Soares, Barbara Fasioli, de Carvalho Elita F Urano, Frederico A. Genezini, Paulo S.C. da Silva, Almir O. Neto, Delvonei A. Andrade

**Affiliations:** † Instituto de Pesquisas Energéticas e Nucleares, 119500 IPEN/CNEN-SP. Av. Prof. Lineu Prestes, 2242 Cidade Universitária, CEP, São Paulo, SP 05508-000, Brazil; ‡ Universidade de São Paulo, USP, Av. Prof. Lineu Prestes, 2242 Cidade Universitária, CEP, São Paulo, SP 05508-000, Brazil

## Abstract

This study examines the feasibility of neutron-induced nuclear transmutation for producing indium-doped tin materials using SnCl_2_ as a model system. Neutron irradiation resulted in structural modifications, including morphological changes, lattice expansion, and the formation of indium-containing crystalline phases. Scanning Electron Microscopy (SEM) and Inductively Coupled Plasma Optical Emission Spectroscopy (ICP-OES) confirmed the presence of indium at approximately 0.88 at. % postirradiation, with a uniform distribution across the material. X-ray Diffraction (XRD) and Raman spectroscopy provided additional evidence of structural changes, supporting the successful incorporation of indium into the SnCl_2_ matrix. These results indicate that nuclear transmutation can be used to produce indium-doped tin materials, offering an alternative approach for synthesizing materials relevant to advanced applications. The process utilizes the neutron capture properties of chlorine to control neutron penetration, contributing to the development of materials with specific characteristics.

## Graphical abstract

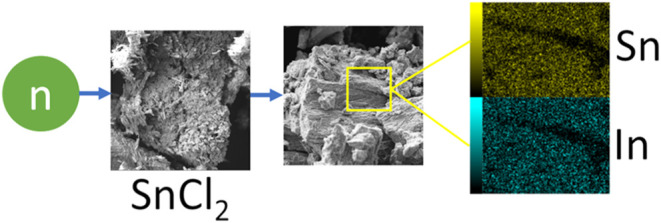

## Introduction

Contemporary technological advancement has been driven by the development of materials with optimized properties for specific applications, ranging from nanoscale manipulation for two-dimensional sheet production to structural defect engineering and chemical doping^1,2^ to achieve desired properties. These strategies have enabled significant advances in electronics, energy, and medicine.^2^ However, the industrial-scale development of these technologies has led to increasing consumption of chemical elements, many with low crustal abundance and complex, costly extraction and purification processes, raising concerns about the long-term availability and sustainability of technologies dependent on these resources.^3^

Indium represents a critical example, being a rare metal that is essential for various advanced technological applications. Its unique properties, including high electrical and optical conductivity, make it indispensable in the manufacture of liquid crystal displays (LCDs), thin-film solar cells (CIGS), and various electronic devices.^4^ The growing demand for these devices has intensified the exploitation of indium reserves, whose scarcity and associated economic and technical challenges pose significant constraints on the continuous development of these technologies.^5^ Given this dependence on a limited resource, investigating viable alternatives to ensure future supply and mitigate environmental and economic impacts related to its scarcity becomes imperative.^4^

In this context, tin emerges as a promising alternative due to its abundance and accessibility. Its chemical and physical properties, including stability, electrical conductivity, corrosion resistance, and ease of acquisition, make it an interesting candidate for technological applications associated or no to indium.^6^ The chemical proximity between tin and indium in the periodic table, combined with advances in material modification techniques, enables the exploration of nuclear transmutation as an alternative route for obtaining indium from tin.

Neutron-induced material modification has proven to be a powerful tool in materials science. Neutron-matter interactions can generate various effects, including point defect formation, atomic displacement in crystal lattices,^7,8^ and alterations in electrical, optical, and catalytic properties.^9,10^ Moreover, neutron capture can trigger nuclear reactions, resulting in unstable isotope formation that decays to another element, a process known as nuclear transmutation, as seen in the conversion of boron to lithium,^9^ tungsten to rhenium, and other metals.^11^ This approach enables postgrowth doping of existing crystals through direct atomic transmutation.^12^

In this scenario, tin chloride (SnCl_2_) emerges as an ideal model material, given its well-defined crystal structure (monoclinic), which does not exhibit other forms unless prepared under special conditions, along with its availability and ease of handling. The transmutation reaction of ^112^Sn, with 0.973% abundance and a thermal neutron capture cross-section of 0.86 (9) and 29 (2) b of resonance integral, led to ^113^Sn, which decays to ^113^In through electron capture and a radioactive nucleus. The presence of chlorine, with its high neutron capture cross-section (∼16.8 × 10^–24^ cm^2^),^13^ further enhances its interaction with incident neutrons, influencing defect production. This work investigates structural changes in tin chloride induced by neutron bombardment and evaluates the possibility of tin-to-indium conversion via neutron capture to create indium-doped tin crystals.

## Results and Discussion

Scanning Electron Microscopy (SEM) analysis revealed significant morphological differences between SnCl_2_ samples before and after bombardment (Figure 1a,b). Initially, the surface exhibited flake-like particles exceeding 200 μm, with pronounced roughness and needle-like crystalline formations, consistent with observations reported by Kamali et al.^14^ Postbombardment, the needle-like formations were largely absent, and the surface appeared smoother, suggesting substantial structural modifications.

**Figure 1:**
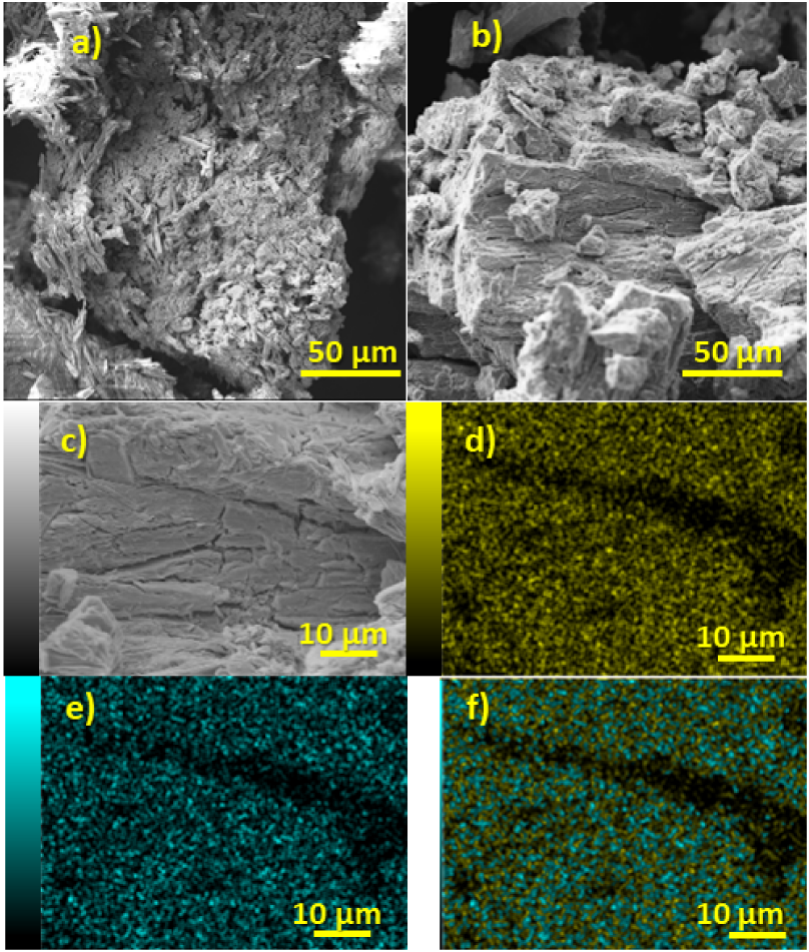
Morphological and elemental characterization: a) SEM micrograph of SnCl_2_; b) SEM micrograph of irradiated SnCl_2_; c) higher magnification SEM micrograph of irradiated SnCl_2_; d) elemental mapping of Sn distribution from the region shown in c); e) elemental mapping of In distribution from the region shown in c); and f) overlaid elemental mapping of Sn and In from the region shown in c).

Elemental analysis, performed using SEM coupled with EDS detection, identified an indium content of approximately 0.90 ± 0.064 at. % (Figure 1d–f). Elemental mapping demonstrated a homogeneous distribution of both Sn and In throughout the composite particles, with In appearing more concentrated on the surface compared to Sn, can be attributed to the distribution and intensity of indium signals at 3.279 keV,^15^ which suggest minimal deviations caused by shielding from superficial atoms. These atoms (e.g., tin) could otherwise absorb or shift the energy of the emitted photons. Additionally, the presence of chlorine atoms in the system acts as a neutron shield due to their high neutron capture cross-section. This characteristic limits the depth to which neutrons can penetrate the grains. The neutron capture cross-section of chlorine is significantly higher than that of tin, with values of 16.8 × 10^–2^ cm^2^ versus 4.9 × 10^–2^ cm^2^.^13^ The transmutation and indium quantities were further validated by ICP analysis, which detected 0.88% ± 0.027%wt In.

X-ray diffraction (Figure 2) analysis revealed predominant peaks characteristic of the monoclinic SnCl_2_·nH_2_O system (JCPDS #75–2033) in both samples. The irradiated sample exhibited additional peaks at 2θ ≈ 18.5°, 26.8°, 30.8°, and 34.9°, corresponding to indium tin chloride (JCPDS #89–833). Further peaks at 2θ ≈ 22.5°, 41.7°, and 49.9° were attributed to indium chloride (JCPDS #26–767), confirming the presence of indium-containing crystalline phases previously detected by EDS.

**Figure 2:**
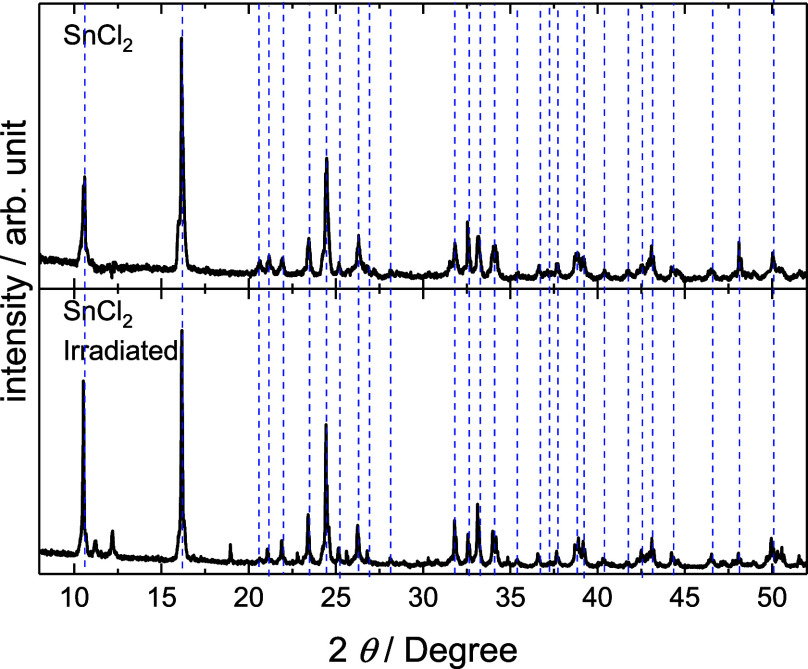
X-ray diffraction patterns of SnCl_2_ and SnCl_2_ irradiated. Dashed vertical lines mark the characteristic 2θ positions for the monoclinic SnCl_2_·nH_2_O phase (JCPDS #75–2033).

Detailed analysis of the main SnCl_2_·nH_2_O phase in the irradiated sample revealed alterations in the relative intensity distribution of crystallographic planes, along with a systematic shift of diffraction peaks toward lower angles, indicating lattice parameter expansion (Table 1). This expansion and consequent increase in unit cell volume are commonly observed in neutron-irradiated materials.^7,8^ The volume expansion can induce structural defects, leading to lattice parameter variations resulting from internal stress accommodation and structural redistribution. These effects are likely enhanced by the displacement of chlorine atoms, which possess a high neutron capture cross-section.

**Table 1:** Lattice Parameters of SnCl_2_ and Irradiated SnCl_2_ Samples

	SnCl_2_		SnCl_2_ Irradiated	
Face	2θ (degree)	d (Å)	2θ (degree)	d (Å)
100	10.584	8.35	10.531	8.40
011	16.136	5.50	16.189	5.48
111	23.412	3.80	23.358	3.81
002	24.448	3.64	24.436	3.64
112	26.322	3.39	26.269	3.38
220	36.604	2.46	36.551	2.46
222	37.684	2.39	37.628	2.39

In the Raman spectrum (Figure 3) for both materials, bands at 100, 127, 217, 243, and 272 cm^–1^,^16^ corresponding to SnCl_2_·nH_2_O, were observed. However, after irradiation, the spectra revealed an increase in the full width at half-maximum (fwhm) of these bands, along with a blueshift. These changes are consequences of the substitution of tin atoms in the structure by (smaller) indium atoms in the lattice (indicated by the blueshift) and crystalline defects caused by vacancies, interstitials, or atomic displacements as a result of a nonuniform distribution of stresses due to the different proportions between tin and indium. This indicates a higher number of accessible microstates, typically associated with more relaxed structures (fwhm) resulting from crystal unit cell volume expansion.^8^ This observation aligns with the XRD results, corroborating the expansion of lattice parameters identified in the irradiated material.

**Figure 3:**
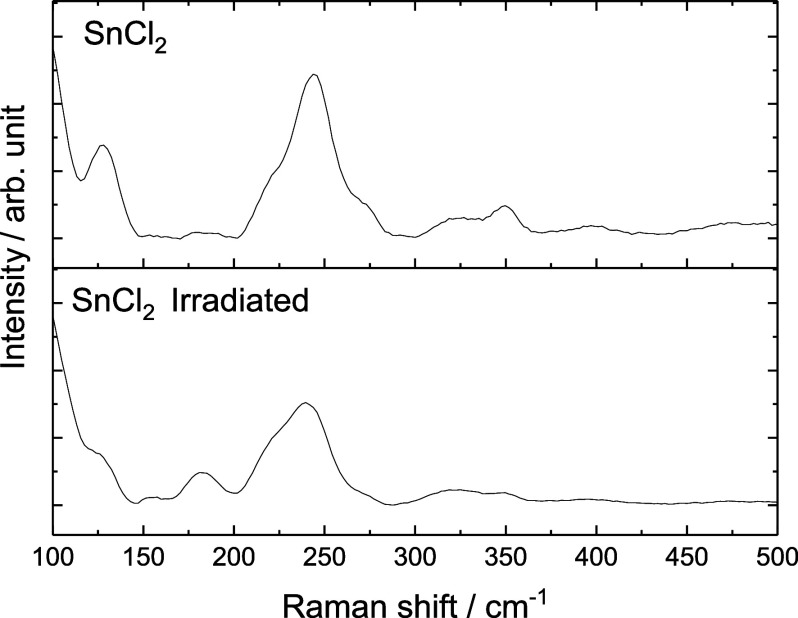
Raman spectra for SnCl_2_ and SnCl_2_ irradiated.

Additionally, well-defined bands at 153 and 174 cm^–1^ were observed in the irradiated sample, potentially corresponding to InCl_3_.^17^ This observation supports the results obtained from other characterization techniques. Neutron irradiation induced defects in the crystal structure of SnCl_2_ and, due to the high chlorine content, additional defects arose from the displacement of chlorine atoms. Additionally, small-scale transmutations of tin to indium were observed.

## Conclusions

This study successfully explored the feasibility of obtaining indium (In)-doped tin (Sn)-containing materials through a neutron-induced nuclear transmutation process using tin chloride (SnCl_2_) as a model material. The results demonstrated significant structural changes in SnCl_2_ after neutron irradiation, including changes in surface morphology, expansion of lattice parameters, and formation of indium-containing crystalline phases. The detection of homogeneous and distributed indium atoms in the material after irradiation, combined with the presence of indium crystalline phases, confirms that the transmutation process was effective (0.88%). Furthermore, the changes in lattice parameters and Raman spectra indicate an irradiation-induced structural reconfiguration, which may influence the physical and chemical properties of the resulting material. Therefore, the tin-to-indium transmutation process offers a promising approach for the production of indium-doped Sn materials. This method not only presents a sustainable alternative to the use of indium but also allows the development of new materials with optimized properties for advanced technological applications.

## Methods

The SnCl_2_·nH_2_O sample was irradiated in the IEA-R1 research reactor at the Nuclear and Energy Research Institute (IPEN), São Paulo, Brazil. The IEA-R1, a pool-type research reactor, operated with a thermal power of 4.5 MW. In this work, the samples were irradiated for 8 h, in a core position with a thermal flux of 2.13 × 10^12^ n·cm^–2^·s^–1^ and an epithermal flux of 2.45 × 10^11^ n·cm^–2^·s^–1^. The flux of fast neutrons is 3.81 × 10^11^ n·cm^–2^·s^–1^.

Morphological characterization was performed using scanning electron microscopy (SEM) on a JSM-IT700HR instrument (JEOL) equipped with a Schottky emission electron gun. Powder X-ray diffraction (XRD) patterns were collected using a MiniFlex II diffractometer (Rigaku) with Cu Kα radiation (λ = 0.154 nm) in the 2θ range of 10° to 90° at a scan speed of 2° min^–1^. Raman spectroscopy was conducted using a MacroRAM Raman spectrometer (Horiba Scientific) equipped with a 785 nm laser excitation source.

Indium content was determined using a SPECTRO BLUE FME26 ICP Optical Emission Spectrometer (AMETEK). Aqueous standards (50 ppm In in 3% HNO_3_) were used for instrument calibration, yielding a calibration curve of i = 2515.65 + 959.83­[In] with R^2^ = 0.997. Auxiliary and coolant argon gas flows were automatically controlled by the instrument.
